# Cubic RSM modeling and multi-criteria evaluation of stainless-steel electrodes for EC of real carwash wastewater

**DOI:** 10.1038/s41598-026-61866-z

**Published:** 2026-07-20

**Authors:** Kholoud Madih, Noha M. Sayed, Rasha H. Ali, Mohamed S. Mahmoud, Hazem Gamal, Rania Osama

**Affiliations:** 1 Chemical Engineering Department, Faculty of Engineering, Minia University, Minia, 61516 Egypt; 2 Department of Engineering and Technology, College of Engineering and Technology, University of Technology and Applied Sciences, Suhar, 311 Oman; 3 Civil Engineering Department, Faculty of Engineering, Minia University, Minia, 61516 Egypt

**Keywords:** Wastewater reuse, EC, Real carwash wastewater, Response surface methodology, Chemistry, Engineering, Environmental sciences, Materials science

## Abstract

This study investigates the treatment of carwash wastewater using the electrocoagulation (EC) process with stainless steel electrodes in a batch electrochemical reactor. The effects of pH (5–9), reaction time (5–50 min), and current (0.5–2 A) on chemical oxygen demand (COD) removal were examined to identify the most effective operating conditions. Response Surface Methodology (RSM) was applied to analyze the interaction between these variables and to develop a predictive model for process optimization. The model achieved a prominent level of accuracy, with an R^2^ value of 0.973, and predicted a maximum COD removal efficiency of 89.82% at pH 9, a current of 2 A, and a treatment time of 27.5 min with a 3.0 cm electrode spacing. Experimental results showed that electrode configuration also played a significant role in system performance. Among the tested designs, the flat mesh electrode achieved the highest COD removal (78.3%), followed by the cylindrical mesh (76.6%), the solid rod (71%), and the flat sheet (59%) under the same initial conditions. In addition, a Multi‑Criteria Decision‑Making (MCDM) analysis was carried out to evaluate the electrodes based on technical performance, energy demand, durability, and cost. The MCDM results supported the experimental findings, identifying the flat mesh electrode as the most suitable option for carwash wastewater treatment using EC. Overall, the study confirms that combining RSM with MCDM provides a reliable approach for optimizing EC systems and selecting the most efficient electrode configuration.

**Supplementary Information:**

The online version contains supplementary material available at 10.1038/s41598-026-61866-z.

## Introduction

Currently, wastewater reuse is increasingly recognized as a viable alternative resource that can significantly alleviate water scarcity^1^. Carwash facilities, in particular, consume substantial amounts of water, requiring careful management and treatment to minimize environmental impacts and preserve surface and groundwater resources. Reported water consumption for washing a single vehicle varies widely, ranging from 45 to 400 L, depending on regional practices and technologies^2^. Typically, an average consumption of 100–200 L per vehicle is considered appropriate, while the International Car Wash Association estimates an average usage of approximately 170 L per medium-sized vehicle^3^.The resulting carwash wastewater is characterized by a complex and variable composition, often containing oils, greases, surfactants, suspended solids, dissolved organic matter, and trace metals^4,5^. With the proliferation of carwash stations and growing concerns about water scarcity, there is a pressing need for efficient, cost-effective treatment technologies that enable water reuse while minimizing environmental pollution.

Among emerging treatment methods, electrocoagulation (EC) has gained considerable attention over the past two decades as a compact and largely chemical-free technology. EC operates through the dissolution of sacrificial metal anodes, generating coagulant species in situ that effectively remove key contaminants such as suspended solids, oil and grease, surfactants, and methylene blue active substances (MBAS)^6,7^.

In contrast to electrocoagulation, which primarily focuses on pollutant removal through physicochemical aggregation, photoelectrochemical advanced oxidation processes offer a more comprehensive level of treatment by generating highly reactive hydroxyl radicals. In particular, the photoelectro-Fenton (PEF) technique has been widely investigated as an effective approach for the mineralization of recalcitrant organic pollutants. Recent studies have demonstrated that PEF processes, especially when combined with engineered photoanodes and optimized reactor configurations, significantly enhance hydroxyl radical production and improve degradation efficiency under light irradiation^8^. Furthermore, the development of semiconductor-based photoanodes, such as TiO₂ and ZnO-immobilized on conductive substrates, has been shown to improve pollutant removal through synergistic interactions between photocatalysis and electro-Fenton reactions^9,10^ . These systems benefit from enhanced charge separation, improved light absorption, and increased stability, resulting in superior degradation performance for persistent organic contaminants under UV or solar irradiation. Such advancements highlight the potential of PEF as a powerful complementary or alternative technology to conventional electrochemical treatment methods, particularly in applications requiring complete mineralization rather than partial removal. Nevertheless, despite the superior oxidation capability of PEF systems, electrocoagulation remains a more economically viable and operationally simpler option, particularly for decentralized wastewater treatment applications such as carwash facilities.

Recent studies have demonstrated the effectiveness of electrocoagulation for treating real carwash wastewater under both batch and continuous configurations addressing both technical performance and economic feasibility^1,11^. For example, Emamjomeh et al.^12^ investigated a hybrid EC-based system incorporating flotation, sedimentation, and filtration. Using response surface methodology (RSM), they optimized key operating parameters (current, electrolysis time, and pH) and evaluated removal efficiencies for COD, turbidity, and MBAS, alongside energy consumption and operational costs. Their work represents one of the more comprehensive techno-economic assessments available and provides a useful benchmark for comparing EC with integrated treatment approaches.

Several studies have focused on the influence of electrode materials on EC performance. Sinno et al.^13^ examined a mixed-electrode configuration (aluminum anode and iron cathode) for treating real carwash greywater, analyzing the removal of turbidity, COD, and total organic carbon under varying pH and reaction times. Similarly, Atiyah and Abdulmajeed^14^ investigated thin aluminum foil electrodes, emphasizing the effects of applied voltage and treatment duration on COD, turbidity, and total dissolved solids (TDS). Muyarip and Aziz^15^ further evaluated aluminum electrodes, studying the impact of current density, pH, and treatment time on simultaneous pollutant removal. In addition, Gönder et al.^16^ compared iron and aluminum electrodes, assessing both treatment efficiency and operating costs under varying process conditions.

Alternative electrode materials have also been explored. Gönder et al.^17^ investigated titanium electrodes using experimental design techniques, while Hoseinzadeh et al. examined multiple electrode types, including stainless steel (SS 304), galvanized iron, and aluminum, in batch EC systems. Although these studies demonstrate promising removal efficiencies, many lack a comprehensive evaluation of energy consumption, cost analysis, and multi-contaminant interactions.

To enhance treatment performance, several researchers have investigated hybrid EC systems. Mirshahghassemi et al.^18^ compared conventional EC with the electro-Fenton process, optimizing parameters and reporting energy consumption, although without a full economic analysis. Moulood and Abdulmajeed^19^ explored Sono-electrocoagulation (Sono-EC), integrating ultrasonic energy with EC, but their work remained limited to laboratory-scale evaluation. Similarly, Gönder et al.^20^ developed an integrated EC–nanofiltration (EC–NF) system, demonstrating the potential for water reuse after sequential treatment.

In addition to hybridization with advanced processes, EC has also been combined with natural and adsorption-based treatments. Latha et al.^21^ investigated the use of Moringa oleifera as a natural coagulant for pretreatment prior to EC, while Rubí-Juárez et al.^22^ coupled EC with granular activated carbon adsorption and conducted equilibrium and kinetic analyses to enhance pollutant removal.

Despite the extensive research on EC, several limitations remain evident. Many studies rely on narrow operational ranges, single-factor experimentation, or conventional second-order optimization models, restricting the robustness and generalizability of their findings. Moreover, literature is heavily dominated by aluminum electrodes, with comparatively limited investigation of stainless-steel systems under systematically optimized conditions.

To address these gaps, the present study investigates the performance of EC using stainless steel (SS) electrodes across an expanded operational domain. A Box–Behnken design (BBD) within a response surface methodology framework is employed to evaluate the effects of current (0.5–2 A), electrolysis time (5–50 min), and pH (5–9) on treatment efficiency. In addition, the influence of electrode geometry is examined under identical operating conditions to identify optimal configurations. This approach enables a more comprehensive and statistically rigorous assessment of EC performance, supporting improved process design and scalability. EC was selected due to its effectiveness as an electrochemical treatment technique. In this process, an applied electric current induces the dissolution of metal anodes, generating metal cations, while hydroxyl ions are produced at the cathode. These species combine to form metal hydroxides that act as coagulants, destabilizing suspended and colloidal particles. Simultaneously, hydrogen gas bubbles generated at the cathode promote the flotation of the formed flocs, thereby enhancing separation via electroflotation.

At the fundamental level, the electrochemical behavior of sacrificial anodes is governed by two competing processes: anodic metal dissolution and oxygen evolution. Metal dissolution is strongly influenced by crystallographic structure and surface properties. For instance, aluminum, with its face-centered cubic (FCC) structure, forms a passive oxide layer that controls dissolution, while iron, with a body-centered cubic (BCC) structure, exhibits more active dissolution pathways due to its lower atomic packing density. Stainless steel, as a multicomponent alloy, exhibits more complex behavior due to the presence of a stable chromium oxide passive layer that limits dissolution while enhancing electrode durability.

Competing with metal dissolution, the oxygen evolution reaction becomes more significant as surface passivation progresses, contributing to floc flotation through oxygen bubble generation. At the cathode, hydrogen evolution produces hydroxyl ions, increasing local pH and promoting the precipitation of metal hydroxides. These coupled electrochemical processes underpin the effectiveness of EC in removing a wide range of contaminants from carwash wastewater.

## Experimental

### Experimental procedures

The typical characteristics of the wastewater used are summarized in Table 1, showing a high organic load (COD 5270 mg/L) and significant suspended solids (TSS 75 mg/L), which are characteristic of untreated carwash effluent. Before introducing the carwash wastewater (0.5 L) into the cell, its conductivity was adjusted by adding a NaCl solution at a concentration of 3 g/L. The concentration of the saline solution added to the wastewater was kept at 3 g/L, which is sufficient to enhance electrical conductivity and improve EC performance, as supported by previous studies^23^. Appropriate acidic or basic solutions were used for pH adjustments according to the RSM conditions. After the experiment was completed, samples were collected and analyzed to determine the COD removal efficiency. The typical characteristics of the wastewater used are summarized in Table 1. Although carwash wastewater contains various pollutants, COD was selected as the primary response because it represents the overall organic and colloidal load and is the most sensitive indicator to electrocoagulation conditions. COD is widely used as the sole optimization parameter in EC-based RSM studies. The inclusion of additional indicators is valuable for future work but does not affect the validity of the present optimization model.

**Table 1:** Characteristics of the wastewater supplied from a local carwash station, Egypt.

Parameter	Value	Unit
pH	6.66	-
Chemical oxygen demand (COD)	5270	mg/L
Total dissolved solids (TDS)	800	mg/L
Total suspended solids (TSS)	75	mg/L
Turbidity	520	NTU
Conductivity	1450	µS/cm
Oil and grease	210	mg/L

### Electrochemical reactor cell set-up

**Fig. 1:**
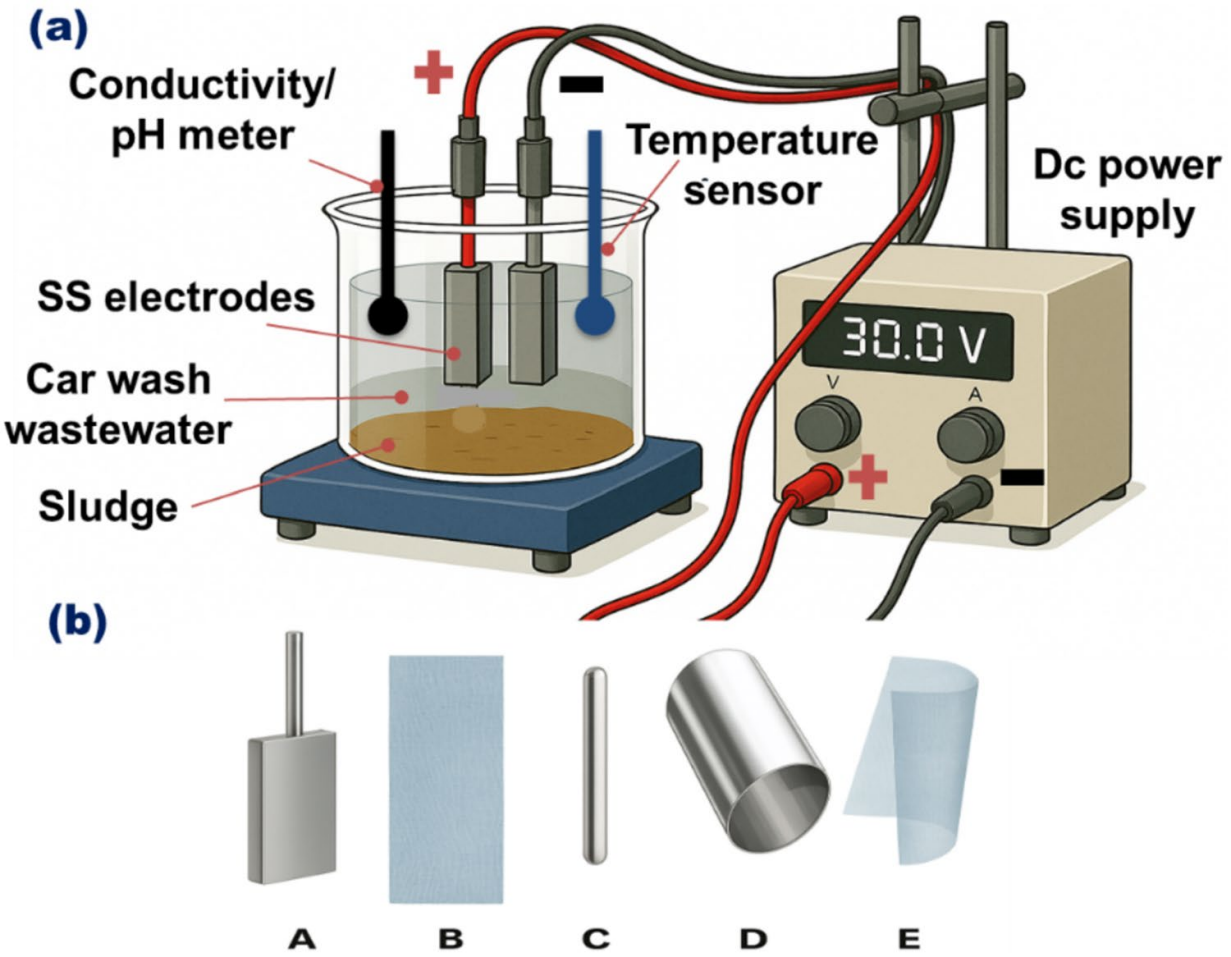
(**a**) Schematic illustration of the EC setup (created with the assistance of AI-based graphic tools). (**b**) Anode electrode configuration used: a) SS plate, b) Flat mesh, c) Solid rod, d) Cylindrical sheet, e) Cylindrical mesh.

The experiments were conducted in a batch electrochemical cell using a 0.5 L cylindrical glass reactor. Two straight rectangular SS plates were employed as the anode and cathode electrodes, with dimensions of 9.0 × 3.0 × 0.2 cm (length × width × thickness) maintaining an inter-electrode distance of 3.0 cm. The effective surface area of the anode was 54 cm^2^, resulting in applied current densities ranging from 9.26 to 37.04 mA/cm^2^. The electrodes were arranged in parallel and placed vertically inside the reactor, maintaining an inter-electrode distance of 3.0 cm. All electrodes were completely submerged in real carwash wastewater that was collected from a local carwash station in Minia Governorate, Egypt. A regulated DC power supply (Thurlby Thandar Instruments, EL302R, UK (has been used to apply the desired voltage across the electrodes. The anode and cathode were connected to the power supply using copper wires, as illustrated in Fig. 1(a)**.** To study the effect of electrode geometry, the anode shape was varied (SS. Flat mesh, SS. Cylindrical mesh, SS. Cylindrical sheet, and SS. Solid rod) as shown in Fig. 1(b)**,** while the cathode was kept as a fixed SS plate.

The energy consumption in the electrocoagulation process was calculated using the standard equation: *E (Wh)* = *V* × *I* × *t,* where V is the applied voltage (V), I is the current (A), and t is the electrolysis time (h). To allow comparison between different electrode configurations, the energy consumption was normalized to volume basis and expressed as kWh/m^3^ according to:

E (kWh/m^3^) = (V × I × t) / Volume, where Volume is the treated wastewater volume (m^3^).

In this study, the corresponding energy values were scaled according to reactor volume (0.5 L) to obtain specific energy consumption. All experiments were conducted under controlled laboratory conditions, and the calculated energy values represent theoretical electrical consumption without accounting for additional system losses.

### Experimental design

RSM encompasses mathematical and statistical techniques that are designed to plan experiments, construct predictive models, evaluate the effects of key variables, and determine optimal operating conditions^24^. In this study, the impact of the most significant operating variables—specifically, current (0.5–2 A), EC time (5–50 min), and pH (5–9)—on removal efficiency is systematically examined using a Box-Behnken design (BBD). The design matrix for the independent variables is listed in Table 2. To assess the significance of all independent factors on the responses studied, a set of 17 random runs of experiments was suggested using the BBD in Design‑Expert® 13 statistical software.

**Table 2:** Independent variables used in the BBD approach.

	**Unit**		**Levels**	
**Factors**		**Minimum (-1)**	**Centre (0)**	**Maximum (+ 1)**
pH	-	5	7	9
Current	[A]	0.5	1.25	2
Time	[min]	5	27.5	50

## Results and discussion

### RSM modeling

Table 3 presents the design matrix for the independent variables. Seventeen randomized experimental runs were conducted to evaluate the significance of each independent factor on the measured responses. The experimental results were fitted to a cubic model to derive the corresponding regression equation. The coded equation obtained by BBD modeling is described by Eq. (1):

1 $$\begin{aligned}{(E)}^{3}&=5.517\times {10}^{5}+33563.31\times\,A+32606.86\times B-9738.68\times\\&C-13710.27\times AC+61431.61\times\,BC-67546.95\times\\&{A}^{2}-41812.18\times {B}^{2}+1.173*{10}^{5}\times {C}^{2}\end{aligned}$$

where E, A, B, and C are the removal efficiency, time, current, and pH, respectively.

**Table 3:** DOE matrix, levels, and response using the BBD.

	Coded level of variables			Actual level of variables			COD removal efficiency (%)
Run	C	A	B	pH	Time(min)	Current(A)	SS electrode
1	-1	0	-1	5	27.5	0.5	86.73
2	1	1	0	9	50	1.25	83.94
3	0	0	0	7	27.5	1.25	81.59
4	1	-1	0	9	5	1.25	82.94
5	0	1	-1	7	50	0.5	76.45
6	-1	1	0	5	50	1.25	87.05
7	1	0	-1	9	27.5	0.5	81.01
8	0	-1	-1	7	5	0.5	72.29
9	0	0	0	7	27.5	1.25	81.02
10	0	0	0	7	27.5	1.25	81.59
11	0	0	0	7	27.5	1.25	82.92
12	0	-1	1	7	5	2	74.95
13	-1	0	1	5	27.5	2	84.34
14	0	1	1	7	50	2	80.60
15	1	0	1	9	27.5	2	89.82
16	0	0	0	7	27.5	1.25	82.92
17	-1	-1	0	5	5	1.25	83.58

Table 4 presents the relevant statistical parameters. The determination coefficient (R^2^), adjusted R^2^, and predicted R^2^ for the models, calculated using ANOVA, are 97.31%, 94.61%, and 78.6%, respectively. The R^2^-Value quantifies the proportion of the variability in COD removal efficiency (%) attributable to initial pH (C), time (A), current (B), and their interactions. The coefficient of determination demonstrates that only 2.45% of the total variation is not accounted for by the models. The reduced predicted R^2^ is primarily due to the high model order relative to the experimental size, rather than a flaw in the model structure. Additionally, the model’s purpose is to support optimization within the studied domain, for which the statistical diagnostics—including ANOVA significance, non-significant Lack-of-Fit, and high adjusted R^2^—remain sound. Moreover, the experimental results were fitted to a cubic model. Because the response was analyzed as (E)^3^ to maximize the correlation coefficient, the statistical parameters in Table 4 reflect this cubic scale. For the raw experimental data, the mean efficiency across the 17 runs was 76.18%, with a maximum of 89.82%.

**Table 4:** Statistical parameters for COD removal efficiency.

Response	R^2^	Adjusted R^2^	Predicted R^2^	Adeq precision	S.D	Mean	C.V.%
COD removal efficiency	0.9731	0.9461	0.786	23.162	19,896.5	5.5E + 05	3.58

Note: S.D. and Mean values are expressed in terms of the transformed response (E)^3^ used for model optimization. The mean COD removal efficiency in raw percentage is 76.18%. effective anode area: 54 cm^2^, current density range: 9.26—37.04 mA/cm^2^, inter-electrode Distance: 3.0 cm.

Table 5 summarizes the ANOVA results for the suggested cubic model describing COD removal efficiency. The model demonstrates high statistical significance, as evidenced by an F-value of 36.13 and a p-value of less than 0.0001. These results indicate that the cubic model explains a substantial proportion of the variability in COD removal efficiency and is appropriate for predicting responses within the experimental domain. The linear terms A-Time (F = 22.76, P = 0.0014) and B-Current (F = 21.49, P = 0.0017) had significant effects on COD removal efficiency (P < 0.05), whereas C-pH was not significant (F = 1.92, P = 0.2036). The interaction between B-Current and C-pH (BC) was highly significant (F = 38.13, P = 0.0003), indicating that the effect of B-Current on COD removal efficiency is dependent on the C-pH level. In contrast, the interaction between A-Time and C-pH (AC) was not significant (F = 1.90, P = 0.2055), suggesting these factors act independently. All quadratic terms (A^2^, B^2^, C^2^) were highly significant: A^2^ (F = 48.53, P = 0.0001), B^2^ (F = 18.59, P = 0.0026), and C^2^ (F = 146.34, P < 0.0001), confirming the presence of significant non-linear relationships with COD removal efficiency. The Lack of Fit F-value was 1.60, with a corresponding P-value of 0.329. Because this P-value exceeds 0.05, the lack of fit is not statistically significant. This result demonstrates that the model fits the experimental data well and adequately represents the relationship between the factors and the response variable.

**Table 5:** Statistical parameters for COD removal efficiency.

**Source**	**Sum of squares**	**DoF**	**Mean square**	**F-value**	**P-value**	
**Model**	1.14E + 11	8	1.43E + 10	36.13	< 0.0001	Significant
A-Time	9.01E + 09	1	9.01E + 09	22.76	0.0014	
B-Current	8.51E + 09	1	8.51E + 09	21.49	0.0017	
C-pH	7.58E + 08	1	7.58E + 08	1.92	0.2036	
AC	7.52E + 08	1	7.52E + 08	1.90	0.2055	
BC	1.51E + 10	1	1.51E + 10	38.13	0.0003	
A^2^	1.92E + 10	1	1.92E + 10	48.53	0.0001	
B^2^	7.36E + 09	1	7.36E + 09	18.59	0.0026	
C^2^	5.79E + 10	1	5.79E + 10	146.34	< 0.0001	
Residual	3.17E + 09	8	3.96E + 08			
Lack of fit	1.95E + 09	4	4.87E + 08	1.60	0.329	not significant
Pure error	1.22E + 09	4	3.04E + 08			
Cor total	1.18E + 11	16				

### Effect of independent parameters

**Fig. 2:**
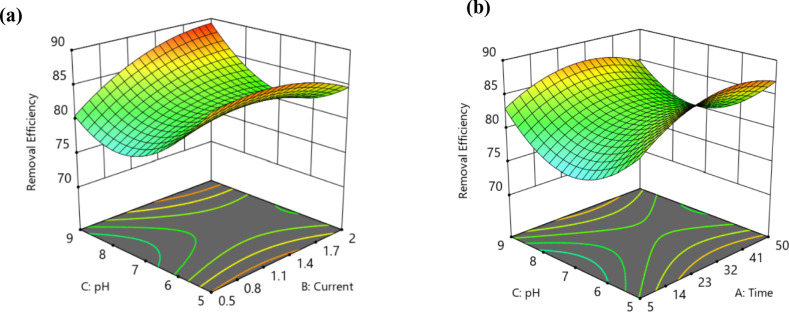
3-D graph of (**a**) pH and current, (**b**) pH and time interactions affect COD removal efficiency.

Figure 2(a) presents a 3D response surface plot which illustrates the interactive effects of current (labeled as B) and pH (labeled as C) on the COD removal Efficiency.

The plot indicates a significant interaction between current and pH. The highest removal efficiency (> 90%) is achieved at the maximum current level (2.0) combined with a low pH value (5–6). As the pH increases at this high current, efficiency declines substantially. Conversely, at low current levels (≈ 0.5), the removal efficiency is relatively high (≈ 80–85%) and exhibits minimal dependence on pH across the tested range. The shape of the surface plot signifies a complex, non-linear relationship between the variables. The results demonstrate that the optimal conditions for maximizing removal efficiency within the studied experimental range are high current and acidic to neutral pH. The strong interaction effect suggests that the mechanism driving the removal process is extremely sensitive to pH, particularly under high-current conditions. Operating at high current in an alkaline environment (pH > 8) appears to be detrimental to the process’s efficiency. The impact of pH on COD removal efficiency was demonstrated in various previous studies, and a substantial effect was observed by^25,26^. Moreover, in the electro-oxidation process using stainless steel (SS) electrodes at pH greater than 6.5, total organic carbon (TOC) removal was significant due to the oxidation of chloride ions to chlorine gas and the formation of hypochlorite, which has a high oxidation potential, increasing the rapidity of the TOC removal^27^, which enhances COD removal efficiency.

Figure 2(b) illustrates a significant interaction between time and pH, as evidenced by a pronounced saddle point at the center of the design space. At shorter time intervals (Time ≈ 5 min), removal efficiency increases with pH, reaching a maximum of approximately 90% at pH 9. As demonstrated by N. Galvão et al.^28^, the efficiency of pollutant removal improves as the solution conductivity increases and the electrolysis time decreases, resulting in a low-cost treatment process. Conversely, at longer time durations (Time ≈ 50 min), the highest efficiency is observed at a low pH of 5, and it decreases as the pH increases. The lowest efficiency is observed at intermediate values for both Time (≈ 25–35 min) and pH (≈ 6–8). Additionally, Fig. 2(a) represents a two-factor slice at a fixed time (27.5 min) and therefore shows a local trend. The RSM optimizer, in contrast, evaluates the full cubic model across all factors simultaneously, leading to the identification of the global optimum at pH 9, 2 A, and 27.5 min.

**Fig. 3:**
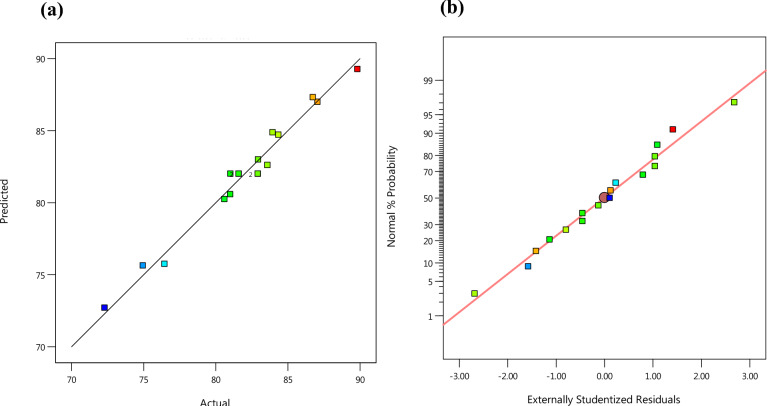
(**a**) Predicted versus actual value of COD removal efficiency, (**b**) Normal Probability Plot of Externally Studentized Residuals of COD removal efficiency.

Figure 3(a) illustrates a close agreement between the predicted and experimental data points, yielding a high coefficient of determination (R^2^). This finding indicates that the model accurately represents the system’s behavior. Consequently, the model’s validity and robustness are supported, confirming its suitability for predicting COD removal efficiency within the examined parameter space**. **Figure 3(b) presents the Normal Probability Plot of Externally Studentized Residuals, which demonstrates that the residuals from the statistical model applied to the removal efficiency data are approximately normally distributed.

### Influence of electrode geometry on COD reduction during EC

**Fig. 4:**
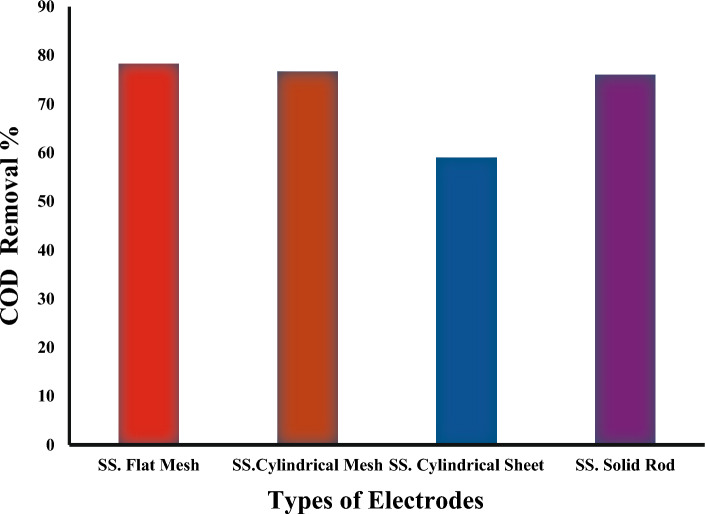
Effect of electrode geometry on COD removal.

Figure 4 shows % COD removal vs electrode geometry. It indicates that electrode geometry plays a key role in COD removal during EC. To evaluate the influence of electrode geometry, tests were performed using the four configurations under the center point conditions identified in the experimental design (pH 7, current 1.25 A, and reaction time 27.5 min). As shown in Fig. 4, the flat mesh electrode achieved the best efficiency (78.3%), followed by the cylindrical mesh (76.6%). This improved effect is primarily attributed to the broader effective body surface area of mesh anodes, which promotes coagulant production and bubble formation. This behavior is consistent with previous studies reporting enhanced EC performance with mesh-type electrodes due to improved hydrodynamics and effective electrode surface utilization. For instance, a similar result regarding the advantage of mesh surfaces was reported by Al‐Qwairi, et al.^29^. The solid rod electrode performed reasonably (71%), and this is consistent with findings from earlier works that the geometry of the electrode and surface area are key factors affecting EC efficiency. The plate sheet electrode had the lowest (59%) pollutant removal since it has a limited active surface area, resulting in reduced coagulant production and weaker pollutant destabilization. The superior performance of mesh-type electrodes can be attributed not only to their larger effective surface area, but also to alleviating the mass transfer and hydrodynamic limitations. The open structure enhances fluid circulation and reduces diffusion limitations, allowing more efficient transport of contaminants toward reactive sites. In addition, the mesh geometry promotes a more uniform current distribution compared to compact electrodes, resulting in consistent anodic dissolution and floc generation.

### Multi-criteria optimization analysis: the potential parameters for car wash water treatment

**Fig. 5:**
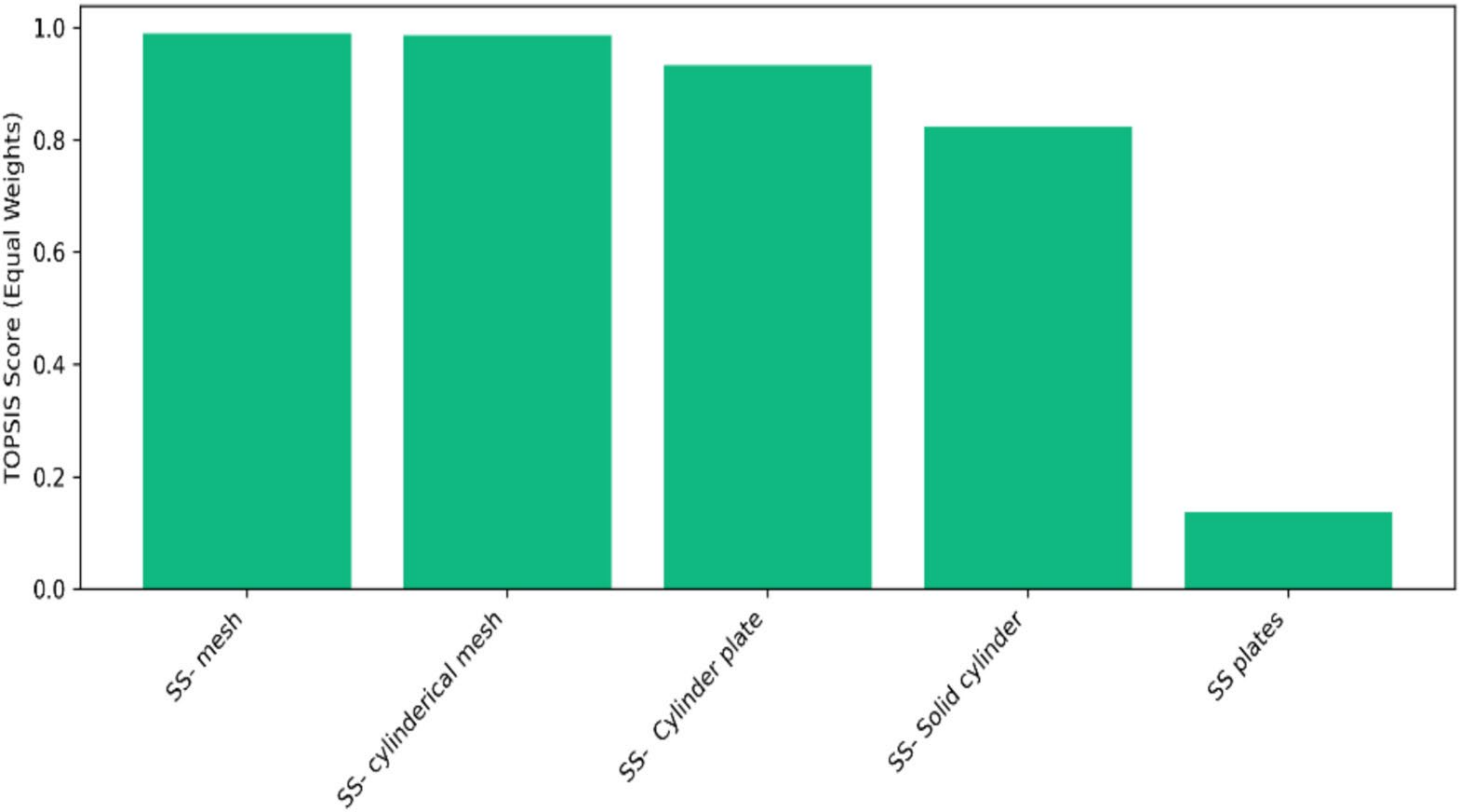
TOPSIS scores SS electrode alternatives under equal weights (Efficiency ↑, Energy & Total Cost ↓).

The EC performance is typically evaluated using several approaches, as operational decisions must balance treatment efficacy, energy requirements, and operating costs. Multiple‑Criteria Decision Making (MCDM)^30^ offers a transparent, quantitative framework to reconcile benefit‑type criteria (e.g., higher COD removal) with cost‑type criteria (to get lower energy and lower total cost). Among MCDM methods, the Technique for Order Preference by Similarity to Ideal Solution (TOPSIS)^31^ is widely used because it ranks alternatives by geometric closeness to an ideal best (maximum efficiency, minimum energy/cost) and an ideal worst solution, after normalization and weighting of the criteria. This ensures reproducible, objective decisions and enables sensitivity analyses of weight schemes. The Decision goal is to identify the most suitable stainless‑steel electrode configuration for car‑wash wastewater EC based on our experiments. The alternatives are SS plates; SS mesh; SS cylindrical mesh; SS cylinder plate; SS solid cylinder. The Criteria studied where: C1—COD removal efficiency (benefit); C2—Energy (Wh/run; cost); C3—Total operating cost (USD/ run; cost). The economic analysis was conducted to evaluate the feasibility of the EC process for carwash wastewater treatment at a representative scale. The economic inputs were restricted to Direct Operating Costs (DOC), specifically focusing on electrical energy consumption and electrode mass loss, as these represent the primary technical cost drivers. The electricity tariff was standardized at 0.10 USD/kWh, and the market price for stainless steel electrodes was estimated at 3.50 USD/kg. To ensure the results are scalable and comparable with other studies, all costs were normalized and reported as USD per cubic meter (USD/m^3^) of treated effluent. Labor and fixed maintenance costs were excluded from this calculation to maintain a focus on the intrinsic performance of the electrochemical reactor across different geographical regions. In addition to electricity consumption and electrode material cost, other cost components like sludge generation and electrolyte consumption are recognized as part of the overall EC operating cost. However, in the present study, the economic evaluation was limited to the primary technical cost drivers (energy and electrode consumption) to enable a controlled comparison between electrode configurations under identical conditions. Sludge production and disposal costs are highly dependent on wastewater characteristics and post-treatment handling strategies and thus were not included in this analysis. Similarly, electrolyte consumption (NaCl) was assumed constant across all experimental runs and therefore omitted from the comparative cost evaluation. Reactor volume = 0.5 L, thus specific energy (kWh/m^3^) = 4 × Wh/run. All criteria were first normalized using vector normalization to remove differences in scale and units. Equal weights (1/3 for each criterion) were assigned to COD removal efficiency, energy consumption, and operating cost to ensure an unbiased baseline evaluation. The TOPSIS algorithm was then applied by constructing the ideal best and ideal worst solutions, followed by calculating the closeness coefficient for each alternative to determine its relative performance. A sensitivity check, in which uniform labor-related overheads were added to all alternatives, showed no change in the ranking, confirming the robustness of the results. Computational data and visualizations were prepared using standard software suites. AI tools (Microsoft M365 Copilot) were employed exclusively for editorial refinement and formatting assistance. All technical data, including TOPSIS optimization results, were manually calculated and rigorously verified by the authors to ensure credibility. The analysis tables (supplementary S-1) provide a complete audit trail. Mesh‑based geometries outperformed other stainless‑steel configurations. Under equal weights, the SS flat mesh ranked first, followed by the SS cylindrical mesh, SS cylindrical plate, SS solid cylinder, and SS plates (as summarized in Table 6). The separation in ranking is driven primarily by the normalized energy consumption (kWh/m^3^). While the SS plates achieved a comparable mean COD removal efficiency 76.18%, their specific energy consumption 11.00 kWh/m^3^ was significantly higher than that of the mesh geometries 2.66–2.75 kWh/m^3^, which heavily penalized the plate configuration on the energy and cost criteria. The MCDM analysis via TOPSIS provided a transparent, quantitative basis for selecting electrode geometries by simultaneously accounting for removal efficiency and DOC. As shown in the scale-up analysis, the operating costs were governed mainly by electrochemical geometry and its associated ohmic resistance rather than fixed overheads. The SS flat mesh demonstrated the most favorable economic profile with an estimated operating cost of 1.72 USD/m^3^. For future industrial implementation, while incorporating capital expenditure (CAPEX) amortization and fluctuating utility tariffs will further refine the economics, the significant energy savings provided by the mesh geometry suggest it remains the most viable candidate for large-scale carwash wastewater treatment. Moreover, sludge generation and its management were not addressed in this study and are recommended as important aspects for future investigation, particularly for large-scale applications. Likewise, further investigation of electrode surface properties, corrosion behavior, and passivation effects using advanced characterization techniques is recommended for future studies to provide deeper insight into material–performance relationships (Fig. 5).

**Table 6:** TOPSIS ranking results for SS electrode alternatives based on efficiency, energy use, specific energy consumption, and total operating cost.

Alternative	Efficiency mean (%)	Energy (Wh)	Specific energy (kWh/m^3^)	Mean total cost (USD/m^3^)	TOPSIS score	Rank
SS flat mesh	78.23	1.375	2.75	1.72	0.989	1
SS cylindrical mesh	76.66	1.329	2.66	1.66	0.986	2
SS cylindrical plate	76.03	1.604	3.21	2.01	0.932	3
SS solid cylinder	58.99	1.696	3.39	2.12	0.823	4
SS plates (RSM Avg)	76.18	5.497	10.99	6.87	0.136	5

## Conclusions

The EC treatment of carwash wastewater using stainless-steel electrodes demonstrated strong potential for improving water quality under optimized operational conditions. Analysis of the experimental data using a Box–Behnken Design enabled the development of a cubic predictive model capable of accurately estimating COD removal performance. The model showed excellent agreement with the experimental results, achieving an R^2^ value of 97.31%, and predicted a maximum COD removal efficiency of 89.82% at pH 9, a current of 2 A, and a reaction time of 27.5 min when using stainless-steel electrodes. These findings confirm both the reliability and predictive strength of the proposed model for optimization and process control. Performance comparison among various stainless-steel electrode configurations revealed noticeable differences in treatment efficiency. The flat mesh electrode achieved the highest experimental COD removal efficiency (78.3%), followed by the cylindrical mesh (76.6%), the solid rod (71%), and finally the flat sheet electrode (59%). However, efficiency alone does not fully represent the overall suitability of an electrode for practical applications. To provide a more comprehensive evaluation, a multi-criteria decision-making (MCDM) approach using TOPSIS was applied, integrating removal efficiency, energy consumption, specific energy use, and total operating cost. The results showed that the flat mesh electrode ranked first, confirming its superior overall performance. The cylindrical mesh electrode followed as the second-best option, while the solid cylinder and flat sheet configurations ranked lower due to reduced efficiency and higher specific energy requirements. The findings highlight that stainless-steel mesh electrodes, particularly the flat mesh type, offer the most balanced and effective option for carwash wastewater treatment, supporting both operational efficiency and cost-effectiveness.

## Supplementary Information

Supplementary Information 1.

Supplementary Information 2.

## Data Availability

The datasets used and/or analysed during the current study available from the corresponding author on reasonable request.
