# Supplementary material for: Cubic RSM modeling and multi-criteria evaluation of stainless-steel electrodes for EC of real carwash wastewater
Source: Sci Rep. 2026 Jul 20;16:22670. doi: 10.1038/s41598-026-61866-z (PMC13385899; doi:10.1038/s41598-026-61866-z)
Supplement: Supplementary file 2 — Supplementary Information 2. [file 41598_2026_61866_MOESM2_ESM.docx]

**Supplementary S-1: Table of experimental and collected data for EC of carwash wastewater**

| **No** | **COD car wash water before mg/L** | **EC Current, A** | **EC Potential V** | **Temp, oC** | **pH** | **Distance between electrodes, cm** | **Conductivity µS/cm** | **Time of treatment, min** | **electrode material** | **COD after treatment** | **% COD removal** | **Ref.** |
| --- | --- | --- | --- | --- | --- | --- | --- | --- | --- | --- | --- | --- |
| 1 | 5270 | 1.25 | 5.45 | 25 | 7 | 3 | - | 27.5 | SS plates | 1060 | 79.88 | This work |
| 2 | 5270 | 0.5 | 5 | 25 | 7 | 3 | - | 50 | SS plates | 1241 | 76.45 |  |
| 3 | 5270 | 2 | 15.5 | 25 | 7 | 3 | - | 50 | SS plates | 1022 | 80.61 |  |
| 4 | 5270 | 0.5 | 3.32 | 25 | 7 | 3 | - | 5 | SS plates | 1460 | 72.29 |  |
| 5 | 5270 | 2 | 15.5 | 25 | 7 | 3 | - | 5 | SS plates | 1320 | 74.95 |  |
| 6 | 5270 | 1.25 | 6 | 25 | 7 | 3 | - | 27.5 | SS plates | 1000 | 81.02 |  |
| 7 | 5270 | 1.25 | 5 | 25 | 7 | 3 | - | 27.5 | SS plates | 970 | 81.59 |  |
| 8 | 5270 | 1.25 | 10 | 25 | 7 | 3 | - | 27.5 | SS plates | 900 | 82.92 |  |
| 9 | 5270 | 1.25 | 7.22 | 25 | 7 | 3 | - | 27.5 | SS plates | 751 | 85.74 |  |
| 10 | 5270 | 2 | 13.5 | 25 | 5 | 3 | - | 27.5 | SS plates | 825 | 84.34 |  |
| 11 | 5270 | 1.25 | 9 | 25 | 5 | 3 | - | 5 | SS plates | 865 | 83.58 |  |
| 12 | 5270 | 0.5 | 5.3 | 25 | 5 | 3 | - | 27.5 | SS plates | 699 | 86.73 |  |
| 13 | 5270 | 1.25 | 10.5 | 25 | 5 | 3 | - | 50 | SS plates | 682 | 87.05 |  |
| 14 | 5270 | 1.25 | 8 | 25 | 5 | 3 | - | 50 | SS plates | 846 | 83.94 |  |
| 15 | 5270 | 1.25 | 7.5 | 25 | 5 | 3 | - | 5 | SS plates | 899 | 82.94 |  |
| 16 | 5270 | 0.5 | 3 | 25 | 5 | 3 | - | 27.5 | SS plates | 1001 | 81.01 |  |
| 17 | 5270 | 2 | 9 | 25 | 5 | 3 | - | 27.5 | SS plates | 5800 | 10.05 |  |
| 18 | 6340 | 0.5 | 6 | 25 | 5 | 3 | - | 27.5 | SS- mesh | 1380 | 78.23 |  |
| 19 | 6340 | 0.5 | 5.8 | 25 | 5 | 3 | - | 27.5 | SS- cylindrical mesh | 1480 | 76.65 |  |
| 20 | 6340 | 0.5 | 7.4 | 25 | 5 | 3 | - | 27.5 | SS- Solid cylinder | 2600 | 58.99 |  |
| 21 | 6340 | 0.5 | 7 | 25 | 5 | 3 | - | 27.5 | SS- Cylinder plate | 1520 | 76.02 |  |
| 22 | 3300 | - | 10 | 25 | 5.5 | 1 | - | 330 | Al (rectangular plates) | - | 90 | [1] |
| 23 | 105 | - | 10 | 25 | 7.3 | 1 | - | 20.3 | Fe (four plates, monopolar) | - | 80.8 | [2] |
| 24 | 1500 | - | 30 | 25 | 3 | 2 | - | 90 | Fe (plates) | - | 94 | [3] |
| 25 | 4369 | - | 30 | 25 |  | 2 | - | 90 | Al (foil electrodes) | - | 97.94 | [4] |
| 26 | 975.1 | - | 10 | 25 | 7.3 | 3 | 755.9 | 90 | Fe anode / SS cathode (plates) | - | 87.5 | [5] |
| 27 | 2032 | - | 30 | Room | 7 | 2 | 1401 | 90 | Aluminum (Foil) | 91 | 95.50% | [6] |
| 28 | 990 | 3 mA/cm² | - | 25 | 8 | - | 1172 | 91 | Iron (Fe) | 119 | 88.00% | [7] |
| 29 | 632 | - | 10 | Room | 7 | - | 403 (TDS) | 30 | Aluminum (Al) | 63 | 90.00% | [8] |
| 30 | 1560 | - | 30 | Room | 3 | 2 | 1400 | 90 | Iron (Fe) | 94 | 94.00% | [9] |
| 31 | 813 | 4.2 mA/cm² | - | Room | 7.3 | - | - | 20.3 | Iron (Fe) | 156 | 80.80% | [10] |
| 32 | 1240 | 105 A/m² | - | Room | 7 | - | 1350 | 60 | Al / Fe | 310 | 75.00% | [11] |
| 33 | 1150 | - | 10 | Room | 5.5 | 1 | 900 | 330 | Aluminum (Al) | 115 | 90.00% | [12] |
| 34 | 480 | 210 A/m² | - | Room | 7 | - | - | 60 | Aluminum (Al) | 77 | 84.00% | [13] |
| 35 | 1050 | 0.5 mA/cm² | - | Room | 5 | - | 1172 | 10 | Titanium (Ti) | 168 | 84.00% | [14] |
| 36 | 580 | 7.5 mA/cm² | - | Room | 7 | 2 | - | 30 | Al-Fe | 365 | 37.00% | [15] |

References

[1] M. Guerreiro Crizel, T. M. Barreto, G. L. Colpani, L. L. Silva, M. A. Fiori, and J. M. Muneron de Mello, “Treatment of Effluents from Vehicle Wash Stations with the Electrocoagulation Process: Proposition of a Predictive Statistical Model for the Estimation of the Efficiency of Chemical Oxygen Demand and Methylene Blue Active Substance Reduction,” ACS ES and T Water, vol. 5, no. 9, pp. 5542–5562, Sep. 2025, doi: 10.1021/acsestwater.5c00547.

[2] S. Mirshahghassemi, B. Aminzadeh, A. Torabian, and K. Afshinnia, “Optimizing electrocoagulation and electro-Fenton process for treating car wash wastewater,” Environmental Health Engineering and Management, vol. 4, no. 1, pp. 37–43, Dec. 2016, doi: 10.15171/ehem.2017.06.

[3] M. J. Mohammadi et al., “Removal of turbidity and organic matter from car wash wastewater by electrocoagulation process,” Desalination Water Treat, vol. 68, pp. 122–128, Mar. 2017, doi: 10.5004/dwt.2017.20319.

[4] I. Mawlood Atiyah and B. Abbas Abdul-Majeed Baghdad, “Carwash Wastewater Treatment by Electrocoagulation Using Aluminum Foil Electrodes,” Journal of Engineering, vol. 25, 2019, doi: 10.31026/j.eng.2019.10.4.

[5] L. Herrera, P. Sigcha, and C. Banchón, “Boosting BOD/COD biodegradability of automobile service stations wastewater by electrocoagulation,” Water Science and Technology, vol. 90, no. 9, pp. 2399–2412, Nov. 2024, doi: 10.2166/wst.2024.357.

[6] I. Mawlood Atiyah and B. Abbas Abdul-Majeed Baghdad, “Carwash Wastewater Treatment by Electrocoagulation Using Aluminum Foil Electrodes,” Journal of Engineering, vol. 25, 2019, doi: 10.31026/j.eng.2019.10.4.

[7] Z. B. Gönder, G. Balcıoğlu, I. Vergili, and Y. Kaya, “Electrochemical treatment of carwash wastewater using Fe and Al electrode: Techno-economic analysis and sludge characterization,” J Environ Manage, vol. 200, pp. 380–390, Sep. 2017, doi: 10.1016/j.jenvman.2017.06.005.

[8] I. Moulood and B. A. Abdul-Majeed, “Treatment of Simulated Carwash Wastewater by Electrocoagulation with Sonic Energy,” Journal of Engineering, vol. 25, no. 9, pp. 30–40, Aug. 2019, doi: 10.31026/j.eng.2019.09.3.

[9] M. J. Mohammadi et al., “Electrocoagulation process to Chemical and Biological Oxygen Demand treatment from carwash grey water in Ahvaz megacity, Iran,” Data Brief, vol. 11, pp. 634–639, Apr. 2017, doi: 10.1016/j.dib.2017.03.006.

[10] S. Mirshahghassemi, B. Aminzadeh, A. Torabian, and K. Afshinnia, “Optimizing electrocoagulation and electro-Fenton process for treating car wash wastewater,” Environmental Health Engineering and Management, vol. 4, no. 1, pp. 37–43, Dec. 2016, doi: 10.15171/ehem.2017.06.

[11] S. O. Ganiyu, E. Vieira dos Santos, E. C. Tossi de Araújo Costa, and C. A. Martínez-Huitle, “Electrochemical advanced oxidation processes (EAOPs) as alternative treatment techniques for carwash wastewater reclamation,” Chemosphere, vol. 211, pp. 998–1006, Nov. 2018, doi: 10.1016/j.chemosphere.2018.08.044.

[12] W. E. Org, H. Rubí-Juárez, C. Barrera-Díaz, I. Linares-Hernández, C. Fall, and B. Bilyeu, “ELECTROCHEMICAL SCIENCE A Combined Electrocoagulation-Electrooxidation Process for Carwash Wastewater Reclamation,” 2015. [Online]. Available: www.electrochemsci.org

[13] A. K. G., M. I., and A.-A. P., “Contemporary Carwash Wastewater Recycling Technologies: A Systematic Literature Review,” World Environment, vol. 11, no. 2, pp. 83–98, Dec. 2021, doi: 10.5923/j.env.20211102.02.

[14] Z. B. Gönder, G. Balcıoğlu, Y. Kaya, and I. Vergili, “Treatment of carwash wastewater by electrocoagulation using Ti electrode: optimization of the operating parameters,” International Journal of Environmental Science and Technology, vol. 16, no. 12, pp. 8041–8052, Dec. 2019, doi: 10.1007/s13762-019-02413-4.

[15] K. , A.-D. Z. , Y. N. Fattah, “reatment of Carwash Greywater Through Electrocoagulation: Effect of Time of Treatment and Inter-electrode Distance,” in Euro-Mediterranean Conference for Environmental Integration, 2024, pp. 19–22.
